# 

**Published:** 1907-09-28

**Authors:** 


### BOOKS RECEIVED.

G. P. Putnam's Sons.
"The Shadow of a Great Rock." By W. R. Lighton.

T. Nelson and Sons.
"Clementina" (Nelson Library).
"If Youth but Knew." By Agnes and Egerton Castle. Nelson Library.
"His Grace." By W. E. Norris, Nelson Library.

Sidney Appleton.
"Minor Medicine." By W. E. Wynter, M.D.

Cassell and Co.
"Insanity." By G. H. Savage, M.D., and E. Goodall, M.D.

The Lippincott Co.
"International Clinics." Vol. III. 17th Series.

Methuen and Co.
"The Care of the Body." By F. Cavanagh, M.D.

The Sanitary Publishing Co.
"The Bacteriological Examination of Disinfectants." By W. Partridge, F.I.C., and C. E. P. Fowler, D.P.H.

The Scientific Press, Ltd.
"The Masseuses' Pocket-book." By Araminta Ross.
"Mental and Sick Nursing." By Robert Jones, M.D.

